# Adsorption-Coupled Oxidation of Single Ag Nanoparticles as Resolved by Stochastic Scanning Electrochemical Microscopy

**DOI:** 10.1021/acs.analchem.5c05592

**Published:** 2026-01-02

**Authors:** Manu Jyothi Ravi, Donald C. Janda, Bagya Sivakumar, Aparajita Adak, Shigeru Amemiya

**Affiliations:** Department of Chemistry, 6614 University of Pittsburgh, 219 Parkman Avenue, Pittsburgh, Pennsylvania 15260, United States

## Abstract

The oxidation of Ag nanoparticles at the ultramicroelectrode (UME) has been extensively studied at the single-particle level for fundamental electrochemistry and electroanalytical sensing. The fast oxidation of a Ag nanoparticle is preceded by the adsorption of the nanoparticle, which can kinetically lower the frequency of amperometric spikes as an important analytical measure. Herein, we combine stochastic amperometry with scanning electrochemical microscopy (SECM) to quantitatively assess the adsorption kinetics of Ag nanoparticles on the Pt UME tip. We developed a theoretical model to simulate the dependence of the collision frequency on the adsorption rate constant and the distance between the tip and an insulating substrate. Experimentally, we confirm the advantage of SECM to determine the adsorption rate constant and diffusion coefficient (or concentration) of nanoparticles when the concentration (or diffusion coefficient) is known. We find that the maximum collision frequency based on the diffusion-limited adsorption of Ag nanoparticles requires a large contact area of a polished and cleaned tip with the nanoparticles. The adsorption of Ag nanoparticles is accelerated by citrate caps, which can be oxidatively chemisorbed on the Pt tip to enable a new covalent mode of nanoparticle–electrode interactions. SECM provides useful mechanistic insights into a deeper understanding of nanoparticle–electrode interactions for superior electrochemical detection.

## Graphical abstract

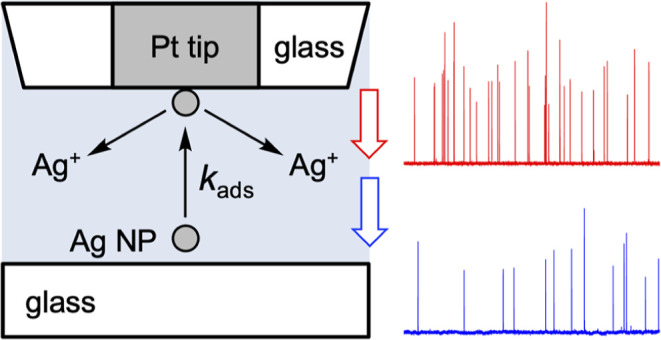

Heterogeneous electron transfer and specific adsorption are coupled in many important electrode reactions of not only small molecules^1–3^ but also nanoparticles.^4^ The adsorption of nanoparticles on the ultramicroelectrode (UME) has been studied electrocatalytically at the single-particle level.^5,6^ The collision of a reactive nanoparticle with an inert UME promotes electrocatalysis of reactant molecules in the solution to amplify an amperometric response.^7^ The diffusion-limited collision of nanoparticles at the disk UME maximizes the frequency, *f*_d_, of the stochastic current responses^8^

1 $$f_{d}=4xDac_{0}$$

where *D* and *c*_0_ are the diffusion coefficient and concentration (particles/volume) of the nanoparticle in the solution, respectively, and *x* is a function of RG^9^ (=*r*_g_/*a*; *r*_g_ and *a* are the outer and inner radii of the UME tip, respectively). Alternatively, the kinetics of nanoparticle adsorption on the UME may limit the collision frequency, *f*_ads_, to yield^8^

2 $$f_{ads}=\pi k_{ads}a^{2}c_{0}$$

where *k*_ads_ is the adsorption rate constant. Recently, both diffusion and adsorption were considered to amperometrically measure *k*_ads_ for citrate-capped Pt nanoparticles at the Au UME modified with alkanethiol monolayers.^10^

Herein, we combine stochastic amperometry with scanning electrochemical microscopy^11,12^ (SECM) to obtain new mechanistic insights into the adsorption-coupled oxidation of single Ag nanoparticles. The oxidation of a single Ag nanoparticle at the UME generates a large number of electrons to yield a measurable current spike.^13^ Stochastic amperometry of single Ag nanoparticles has been extensively studied for fundamental electrochemistry^14^ and electrochemical sensing.^15^ The oxidation of a Ag nanoparticle can be extremely fast at sufficiently positive potentials.^16^ The resultant collision frequency, however, rarely reaches a diffusion limit,^17,18^ thereby indicating a kinetic limit.^19^ Several studies have been reported to increase the collision frequency, which determines sensitivity to the concentration of nanoparticles for sensing applications.^15^ Either UMEs^20–23^ or Ag nanoparticles^24,25^ were chemically modified to enhance noncovalent nanoparticle–electrode interactions for higher collision frequencies. The enhanced frequencies were still below the diffusion limit and were not quantitatively analyzed to yield *k*_ads_. Problematically, the collision frequency is lowered by the adsorption of organic contaminants on the UME^26^ to hamper the measurement of intrinsic adsorption kinetics.

This work is the first to investigate the kinetically controlled adsorption of nanoparticles as SECM probes. Theoretically, we simulate the steady-state current based on the adsorption-coupled oxidation of an ensemble of Ag nanoparticles at the tip to represent the collision frequency of single nanoparticles. Experimentally, we validate the simulated collision frequency, which decreases owing to the hindered diffusion of Ag nanoparticles to the tip as the tip approaches an insulating substrate (Figure 1). We also demonstrate that the negative feedback effect^11,12^ varies with the dimensionless adsorption rate constant, λ_ads_, given by

3 $$\lambda_{ads}=\frac{ak_{ads}}{D}$$

**Figure 1:**
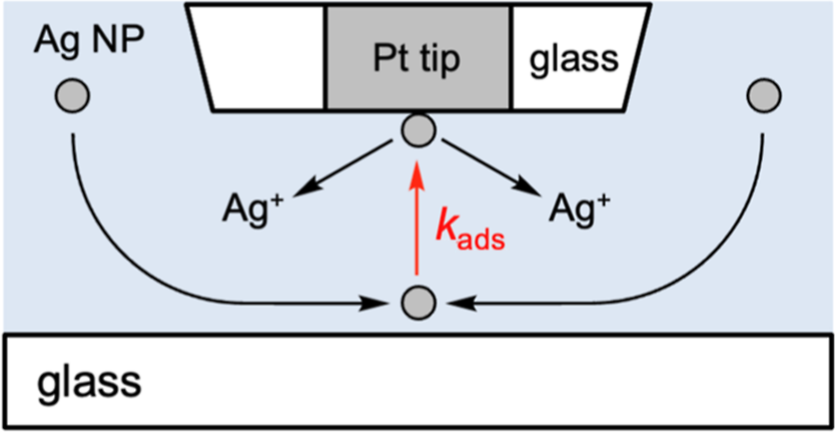
Scheme of adsorption-coupled oxidation of single Ag nanoparticles at the UME tip positioned near a glass substrate by SECM.

Advantageously, SECM allows for the determination of *k*_ads_ and *D* (or *c*_0_) with knowledge of *c*_0_ (or *D*). Previously, SECM was applied to assess the collision frequency of single nanoparticles at the UME tip only under the diffusion limit.^27^ The adsorption of nanoparticles was not considered in other SECM studies of nanoparticles based on stochastic amperometry^28–30^ or steady-state approach curve measurements.^31,32^ By contrast, SECM was applied to resolve the adsorption of small redox molecules from electron transfer at the substrate^33–35^ and more recently at the tip.^36^

Specifically, we find that *k*_ads_ of 40 nm-diameter Ag nanoparticles varies by 4 orders of magnitude from a diffusion limit to a purely kinetic limit. We manifest critical requirements for the diffusion-limited adsorption of citrate-capped Ag nanoparticles at the Pt UME. The nanoparticle solution must be clean enough to minimize the adventitious contamination of the tip.^26^ The adsorption kinetics was compromised by 2 orders of magnitude with the aged stock solution of Ag nanoparticles. Moreover, KCl was used as a supporting electrolyte to facilitate the oxidative dissolution of Ag nanoparticles.^37^ Furthermore, the surface of a Pt UME was roughened on the nanometer scale to increase the area of contact with nanoparticles. By contrast, the adsorption of Ag nanoparticles slowed down by 3 orders of magnitude as the UME tip was flattened by focused-ion-beam (FIB) milling. Remarkably, the adsorption of Ag nanoparticles capped with polyethylene glycol (PEG) was 4 orders of magnitude slower than that of citrate-capped nanoparticles. Citrate can be oxidatively adsorbed on the Pt surface to mediate a new covalent mode of nanoparticle–electrode interactions for superior electrochemical detection.

## Model

We developed a steady-state diffusion-reaction model for SECM (Supporting Information) to simulate the dependence of the collision frequency on the adsorption rate constant, *k*_ads_, and the tip–substrate distance, *d*. Our model assumes that the tip current based on the oxidation of an ensemble of Ag nanoparticles, *i*_T_, is related to the collision frequency, *f*, of single Ag nanoparticles by

4 $$i_{T}=nef$$

where *n* is the number of electrons involved in the oxidation of a Ag nanoparticle and *e* is the elemental charge. The steady-state model is implied in eqs 1 and 2 ^8^ and was validated experimentally when the collision frequency was diffusion-limited.^27^ The steady-state tip current can be simulated by solving the axisymmetric diffusion problem of SECM as represented by a diffusion-limited value in the bulk solution

5 $$i_{T,\infty }=4xneDc_{0}a$$

A combination of eq 5 with eq 4 for *f*_d_ yields eq 1 for any RG.

The current response of the tip positioned in the bulk solution was simulated and converted to a collision frequency (circles in Figure 2) to fit an analytical equation (solid line)

6 $$\frac{f_{\infty }}{f_{d}}=\frac{1}{1+\frac{4x}{\pi \lambda_{ads}}}$$

**Figure 2:**
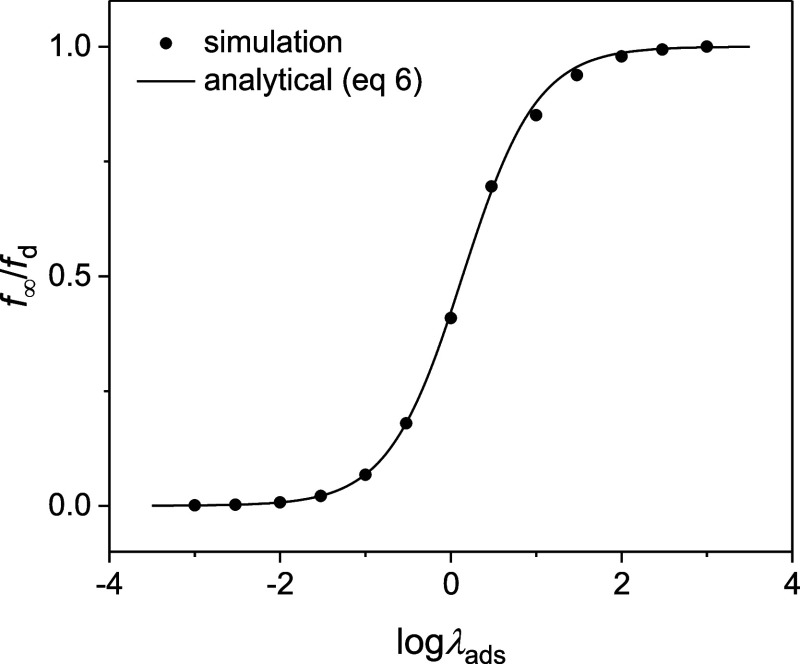
Normalized collision frequencies at the UME in the bulk solution as simulated for various λ_ads_ values under steady states (circles). The solid line represents the analytical expression (eq 6).

Equation 6 was derived for a UME tip with any RG by considering the mixed diffusion and kinetic control of nanoparticle collision^8^ (see Supporting Information). The good agreement validates our model. Both our simulation and eq 6 illustrate a transition from diffusion-limited adsorption to kinetically controlled adsorption, as the normalized adsorption rate constant, λ_ads_, decreases.

We also simulated the dependence of the collision frequency on the tip–substrate distance, i.e., the approach curve, with various λ_ads_ values. Experimentally, the tip–substrate distance is limited to *d*/*a* > 0.1 (solid lines in Figure 3) owing to imperfect alignment between the tip and the substrate.^38^ Negative approach curves with λ_ads_ > 10 were simulated for the diffusion-limited adsorption of a nanoparticle to agree with the corresponding analytical expression.^9^ As λ_ads_ becomes smaller, the approach curve becomes less negative; i.e., the collision frequency becomes less sensitive to the tip–substrate distance. Eventually, a negligible change in the collision frequency is expected with λ_ads_ < 0.01.

**Figure 3:**
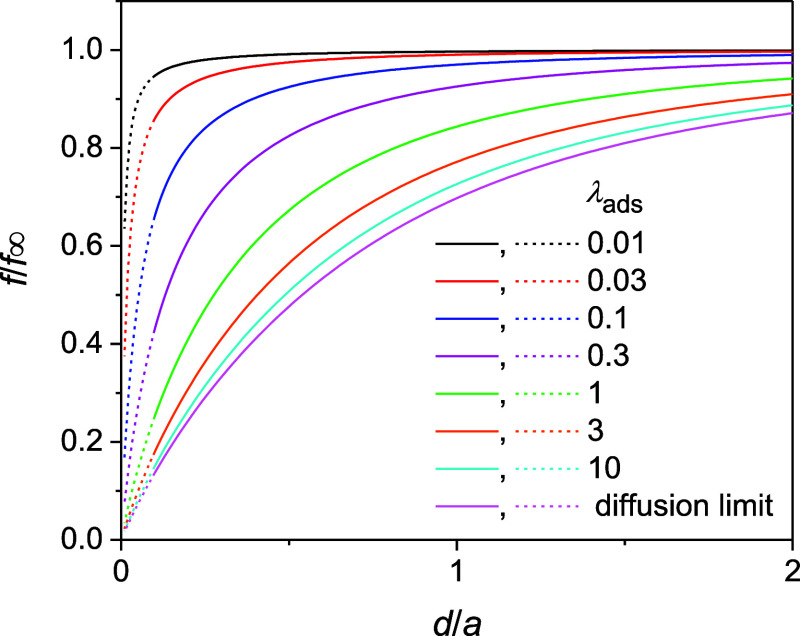
Normalized approach curves based on the collision frequencies at the UME tip positioned at various distances from an insulating substrate as simulated for various λ_ads_ values under steady states (solid and dotted lines for *d*/*a* > 0.1 and <0.1, respectively). The diffusion-limited curves represent the analytical expression of the negative approach curve.^9^

Importantly, the unique approach curve of each λ_ads_ value was obtained by normalizing the distance-dependent collision frequency, *f*, against the collision frequency in the bulk solution, *f*_∞_. Subsequently, the approach curve was independent of the diffusion-limited frequency, *f*_d_, which may not be measurable experimentally and can be obtained from eq 1 only if both *D* and *c*_0_ are known. We demonstrate experimentally the advanced capability of SECM to determine both *k*_ads_ and *D* (or *c*_0_) from an approach curve with knowledge of only *c*_0_ (or *D*). Chronoamperometry has been developed to determine either *c*_0_ from diffusion-limited collision frequencies^39^ or *k*_ads_ from adsorption-controlled frequencies.^10^

Alternatively, single-nanoparticle amperometry and ensemble stripping voltammetry were combined to determine *f*_∞_ and *f*_ads_, respectively, thereby yielding a sticking coefficient, *f*_ads_/*f*_∞_.^19^ In principle, *k*_ads_ and *D* (or *c*_0_) can also be determined from *f*_ads_ and *f*_∞_. The stripping voltammogram, however, demonstrated a single current peak based on the oxidation of many adsorbed Ag nanoparticles to determine only the number of oxidized Ag atoms. The average charges involved in the oxidation of single Ag nanoparticles were approximated to obtain *f*_ads_, which was not used to determine either *k*_ads_ or *D* (or *c*_0_).

## Experimental Section

### Chemicals and Materials

Stock solutions of 30, 40, 50, and 60 nm-diameter citrate-capped Ag nanoparticles (0.02 mg/mL) with 2 mM sodium citrate were purchased from Nanocomposix (San Diego, CA). A 1 mg/mL stock solution of 40 nm-diameter Ag nanoparticles with PEG caps (MW 2000) was obtained from Cytodiagnostics (Burlington, ON, Canada). A Milli-Q IQ 7003 water purification system (EMD Millipore, Billerica, MA) was used to obtain UV-treated deionized ultrapure water (18.2 MΩ·cm) with a total organic carbon of ∼5 ppb.

### SECM Measurements

A home-built SECM instrument^40^ with a patch-clamp amplifier system (Axopatch 200B and Digidata 1550B, Molecular Devices, San Jose, CA) was controlled by using a custom LabVIEW program (National Instruments, Austin, TX). Pt tips of 10 μm and 25 μm diameters were purchased from CH Instruments (Austin, TX). In addition, a 25 μm-diameter Pt tip was prepared by sealing a Pt wire in a pulled glass capillary.^41^ The tip was milled by the focused Ga^+^ beam (30 keV) using a dual-beam instrument (Scios, FEI, Hillsboro, OR) to expose a Pt disk, as imaged by scanning electron microscopy.

A glass SECM cell was used under ultrapure air to minimize adventitious airborne contamination. We employed ultrapure air, which contained O_2_ as used for tip positioning (see Supporting Information). The cell was cleaned in concentrated sulfuric acid at 80 °C for 60 min,^42^ rinsed with ultrapure water, and sonicated in ultrapure water for 30 min immediately before use. The glass cell was purged with ultrapure air (Ultra Zero, Matheson, Irvin, TX) and filled with ultrapure water to dissolve electrolytes before a stock solution of nanoparticles was added. The flow of ultrapure air was maintained above the nanoparticle solution during the SECM measurements. The Pt tip was cleaned in 20 mM KNO_3_ by cyclic voltammetry to dissolve trace Ag and AgCl residues before and after SECM experiments. The Pt tip was further cleaned in piranha solution (a 1:3 mixture of 30% H_2_O_2_ and 95.0–98.0% H_2_SO_4_). Caution: Piranha solution reacts violently with organics and should be handled with extreme care! The piranha-cleaned tip was rinsed with ultrapure water immediately before it was immersed in the nanoparticle solution or stored for future use. The tip was not polished with Al_2_O_3_ particles or diamond pastes, which can contaminate the tip surface.^43^ A Ag/AgCl wire was cleaned with ultrapure water before being immersed in the nanoparticle solution. The Pt tip was positioned near the bottom of the glass cell by measuring an approach curve based on the diffusion-controlled oxygen reduction reaction at the tip (Figure S3).

The tip current response based on the oxidation of single Ag nanoparticles was measured for 180 s at the tip potential of 0.6 V. The low-nA current spikes were measured with a sampling interval of 50 μs by setting a gain, α, of 0.5, a feedback resistor of 500 MΩ (β = 1), and a filter frequency of 1 kHz. We used Clampfit 11.1 (Molecular Devices) to integrate charges under each peak with respect to the background level, which was determined by the average current in the peak-free region. The background level was independent of the tip–substrate distance and was not involved in the determination of the current peak. Specifically, a collision frequency was calculated from the number of peaks that exceeded 10% of the charge based on the complete oxidation of a 40 nm Ag particle (0.31 pC). The threshold of 10% was set to minimize the count of the spikes based on the repetitive oxidation of the same nanoparticles.^16^

## Results and Discussion

### Diffusion-Controlled Oxidation of Ag Nanoparticles

We observed the nearly diffusion-limited collision of citrate-capped Ag particles with diameters of 30, 40, 50, and 60 nm on the 25 μm-diameter polished Pt UME. Here, we illustrate the results of 40 nm-diameter particles, which were employed for the rest of this work. The diffusion limit was calculated by using eq 1 with the diffusion coefficient of the 40 nm-diameter particles as estimated from the Stokes–Einstein equation^16^

7 $$D=\frac{k_{B}T}{6\pi \eta r}$$

where *k*_B_ is the Boltzmann constant (1.381 × 10^–23^ J/K), η is the viscosity of water (0.8937 mPa·s), and *r* is the nanoparticle radius. Equation 7 with *r* = 20 nm gives *D* = 1.2 × 10^–7^ cm^2^/s, which corresponds to *f*_d_ = 19 s^–1^ in eq 1 with *c*_0_ = 47 pM and *a* = 12.5 μm when *x* = 1.08 for RG = 2.5 is considered.^9^ The collision frequency of the citrate-capped Ag nanoparticles was measured with a 25 μm-diameter Pt tip in the bulk solution (top panel of Figure 4A). We obtained *f*_∞_ values of 16 ± 2 s^–1^ (*N* = 10), which are nearly diffusion-controlled. The *f*_∞_/*f*_d_ ratio of 0.84 corresponds to λ_ads_ = 10 (Figure 2), which is equivalent to *k*_ads_ = 1.0 × 10^–3^ cm/s in eq 3. It should be noted that an average nanoparticle diameter of 40 ± 5 nm was determined by transmission electron microscopy (Figure S2). The relative standard deviation (12.5%) of the nanoparticle diameter directly propagates to the diffusion coefficient of the nanoparticles (eq 7) and then to the adsorption rate constant (eq 3).

**Figure 4:**
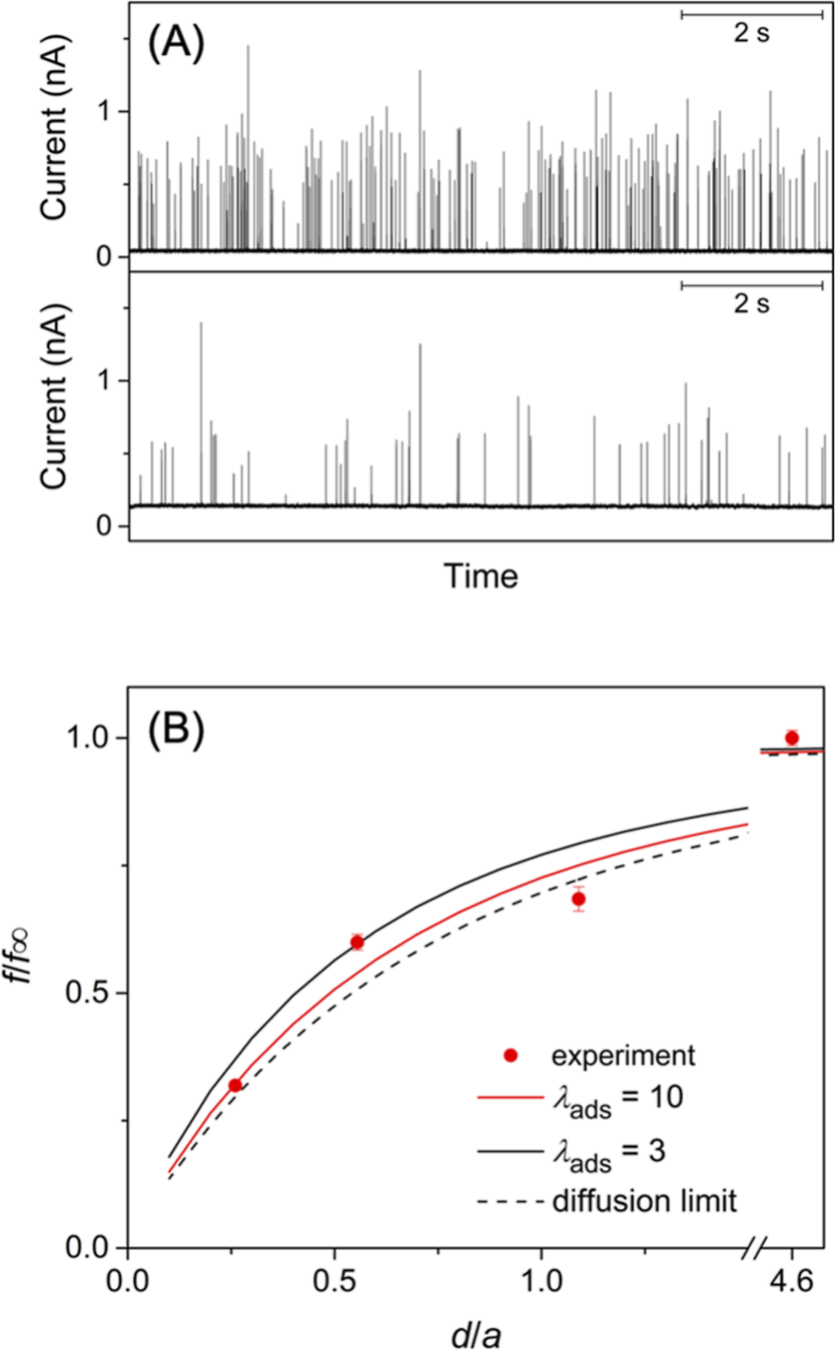
(A) The current response of a 25 μm-diameter Pt UME to the oxidation of 40 nm-diameter citrate-capped Ag nanoparticles in the bulk solution (top) and at *d* = 3.2 μm from the glass substrate (bottom). The solution of 47 pM nanoparticles also contained 20 mM KCl and 2 mM sodium citrate. (B) Experimental collision frequencies at different tip–substrate distances (dots) as compared with theoretical curves (solid and dashed lines).

We employed SECM not only to ensure the diffusion limitation of the collision frequency but also to determine both the adsorption rate constant and the diffusion coefficient of Ag nanoparticles. The collision frequency was lowered as the tip was positioned closer to a glass substrate (the bottom panel of Figure 4A), which hindered the diffusion of nanoparticles to the tip. The experimental dependence of the collision frequency on the tip–substrate distance agreed well with the theoretical dependence to confirm the diffusion limitation (Figure 4B). In this analysis, the collision frequency, *f*, was normalized by that in the bulk solution, *f*_∞_, and plotted against the tip–substrate distance, *d*, normalized by the tip radius, *a*. A good fit was obtained by adjusting only the initial distance of the tip from the substrate to yield λ_ads_ = 10. This rate constant is consistent with the value estimated from eq 6 only by the amperometry of *f*_∞_ (see above). A *f*_d_ value, however, was unmeasurable by amperometry and was calculated from eq 1 with a *D* value estimated from eq 7. By contrast, SECM determined both λ_ads_ and *D* values experimentally and separately. Specifically, *f*_∞_ and λ_ads_ values were determined by SECM and used with eq 6 to calculate a *f*_d_ value. The *f*_d_ value yielded a *D* value of 1.2 × 10^–7^ cm^2^/s from eq 1 with known values of *c*_0_ = 47 pM and *a* = 12.5 μm.

We also found that current spikes are lower at a shorter tip–substrate distance (Figure 4A), which indicates less complete oxidation of Ag nanoparticles. The charges under the spikes were integrated to average at 0.23 ± 0.12 pC (1752 spikes) and 0.19 ± 0.09 pC (442 spikes) in the bulk solution and at 3.2 μm from the glass substrate, respectively. The respective averages correspond to 74 and 61% of the charges based on the complete oxidation of a 40 nm-diameter Ag nanoparticle (0.31 pC). Lower spike currents are attributed to the hindered diffusion of Cl^–^ by the glass substrate. Chloride is needed for the oxidation of Ag nanoparticles adsorbed on the UME to mediate the rate-determining nucleation of AgCl.^37^ Lower current spikes were observed at the tip in the bulk solution when the Cl^–^ concentration was lowered from 20 to 10 mM. By contrast, the hindered diffusion of Cl^–^ by the glass substrate minimally affected the collision frequency controlled by the diffusion of Ag nanoparticles (Figure 4B).

### Adsorption-Controlled Oxidation of Ag Nanoparticles

The adsorption of Ag nanoparticles was kinetically controlled when the stock solution of the nanoparticles was aged to cause adventitious contamination of the Pt tip. The stock solution of nanoparticles was used multiple times in an ambient environment (typically every day for more than a week to prepare a diluted solution) and contaminated with small airborne organic molecules,^44^ which are adsorbed on the UME to lower collision frequencies.^26^ The frequencies were much lower than expected from a change in the nanoparticle concentration in the aged solution. The kinetic effect was more significant with a 10 μm-diameter polished Pt tip because the smaller tip provided a higher mass-transport condition (eq 3). The corresponding diffusion-limited frequency of 3.9 s^–1^ was estimated from eq 1 with *D* = 1.2 × 10^–7^ cm^2^/s and *c*_0_ = 23.5 pM. The experimental frequency of 0.19 ± 0.03 s^–1^ was much lower than the diffusion limit (the top panel of Figure 5A) to yield *f*_∞_/*f*_d_ = 5.0 × 10^–2^. This ratio is equivalent to λ_ads_ = 0.1 (Figure 2), which is still large enough to maintain a diffusional contribution to the measured frequency (Figure 3). This prediction was confirmed experimentally by measuring the collision frequency at the Pt tip positioned near the glass substrate. The collision frequency was lowered as the tip was positioned at 1.1 μm from the glass substrate (bottom panel of Figure 5A). The substrate hindered the diffusion of the nanoparticles to the tip. Current spikes were similarly high near the substrate and in the bulk solution, because less Ag^+^ was generated to consume less Cl^–^ in the tip–substrate gap. The integrated charges under the spikes averaged at 0.22 ± 0.16 pC (47 spikes) and 0.21 ± 0.14 pC (41 spikes) in the bulk solution and at 1.1 μm from the glass substrate, respectively. The respective charges correspond to 71% and 68% of the charges based on the complete oxidation of a 40 nm-diameter Ag nanoparticle (0.31 pC). These charges are very similar to those measured with a fresh nanoparticle solution (Figure 4A). This result indicates that the lower collision frequencies are not due to the aggregation of nanoparticles, which not only lowers collision frequencies but also yields higher charges based on the oxidation of multiple nanoparticles.^45^

**Figure 5:**
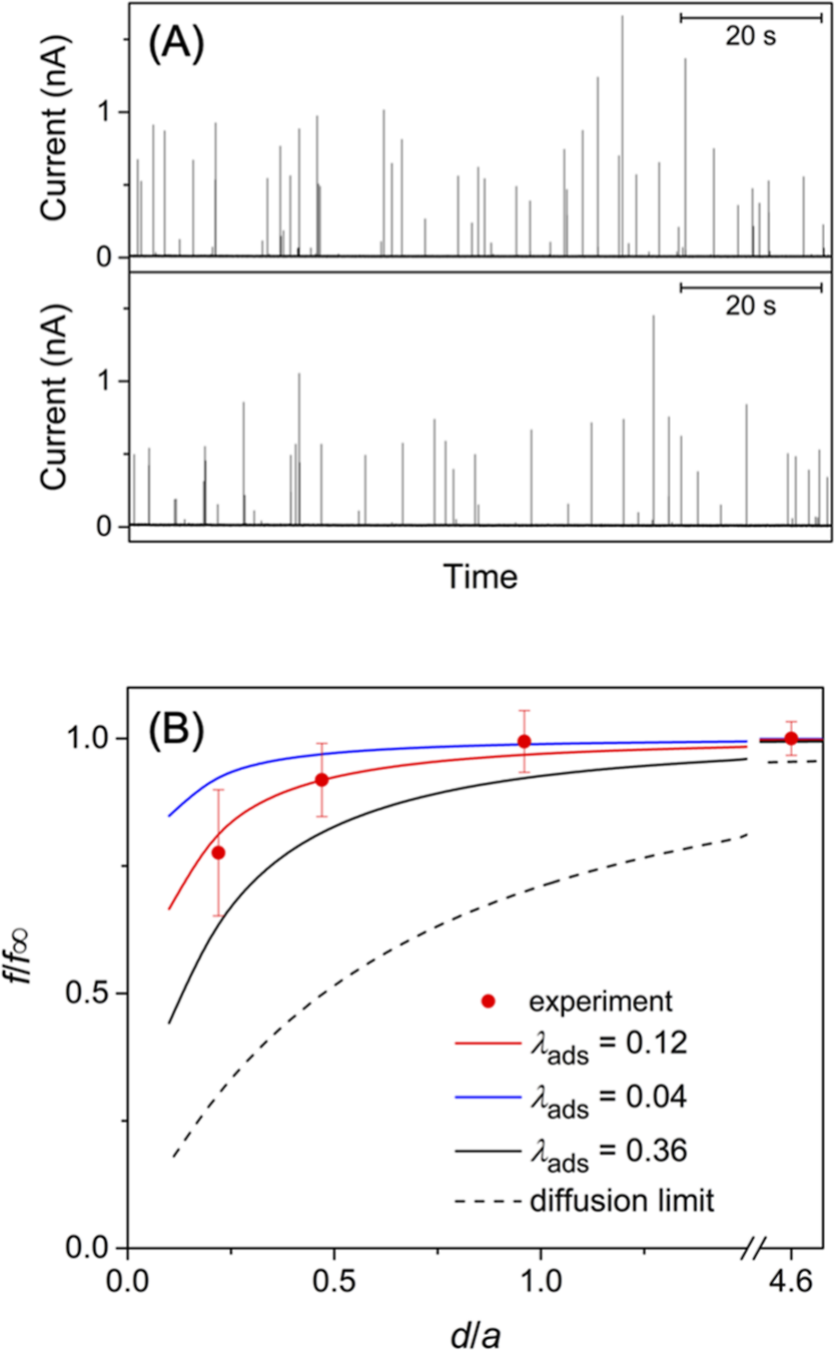
(A) The current response of a 10 μm-diameter Pt UME to the oxidation of 40 nm-diameter citrate-capped Ag nanoparticles in the bulk solution (top) and at *d* = 1.1 μm from the glass substrate (bottom). The solution of 23.5 pM nanoparticles also contained 20 mM KCl and 2 mM sodium citrate. (B) Experimental collision frequencies at different tip–substrate distances as fitted with the theoretical curves limited by adsorption or diffusion (solid and dashed lines, respectively).

The distance-dependent collision frequency was measured experimentally and compared with theoretical approach curves to agree well with a kinetically controlled one (Figure 5B). The λ_ads_ value of 0.12 was determined from the good fit and was similar to the λ_ads_ value of 0.1 as estimated from the *f*_∞_/*f*_d_ value only by amperometry (see above). The respective λ_ads_ values correspond to *k*_ads_ values of 2.9 × 10^–5^ cm/s and 2.4 × 10^–5^ cm/s. The SECM measurement, however, is not redundant and is informative in comparison to the amperometric measurement of only *f*_∞_. The determination of *k*_ads_ by amperometry only in the bulk solution required the diffusion coefficient and bulk concentration of nanoparticles to calculate a *f*_d_ value. The *f*_d_ value was not measurable but was needed for determination of *k*_ads_. By contrast, only the bulk concentration was needed to determine *k*_ads_ and the diffusion coefficient from the SECM measurement. It should be noted that an adsorption-limited collision frequency is amenable to various uncontrollable factors, e.g., adventitious contaminants and AgCl precipitates, thereby causing larger errors (Figure 5B) than those of the diffusion-limited counterpart (Figure 4B).

### Effect of Electrode Surface Roughness

We found that the surface of a Pt UME tip must be roughened to yield a high collision frequency. Nearly diffusion-limited collision frequencies (Figure 4) were obtained using commercial Pt tips, which were mechanically polished to obtain a rough surface (Figure 6A). We also tested a homemade 25 μm-diameter Pt tip, which was milled and flattened by the focused ion beam (FIB) of Ga^+^ (Figure 6B). The flat Pt tip yielded very low collision frequencies of (6 ± 2) × 10^–2^ s^–1^ (*N* = 5) under the same conditions (Figure 6C). The collision frequencies at the FIB-milled tip correspond to λ_ads_ = 3 × 10^–2^ (Figure 2), where the diffusional mass transport of nanoparticles is nearly negligible (Figure 3). The low λ_ads_ value yields a low *k*_ads_ value of 3 × 10^–6^ cm/s in eq 3 to represent a purely adsorption-controlled oxidation of Ag nanoparticles. This collision frequency is 3 orders of magnitude lower than a *k*_ads_ value of 1.0 × 10^–3^ cm/s at the polished tip. This difference is much larger than the difference in the electrochemically active surface areas between polished and FIB-milled tips. Specifically, the electrochemically active surface area of the polished tip was 4.6 times larger than that of the FIB-milled tip, as estimated by cyclic voltammetry of underpotential hydrogen deposition (Figure S4A,B, respectively). The corresponding charge density of 2.1 × 10^–4^ C/cm^2^ for underpotential hydrogen deposition on the milled Pt surface is close to that of 2.4 × 10^–4^ C/cm^2^ on the Pt(111) surface.^46^ A FIB-milled Pt tip was not fouled by contamination with Ga^+^ and efficiently mediated the hydrogen evolution reaction (Figure S4C), which requires the adsorption of hydrogen atoms on the surface Pt atoms.^47^

**Figure 6:**
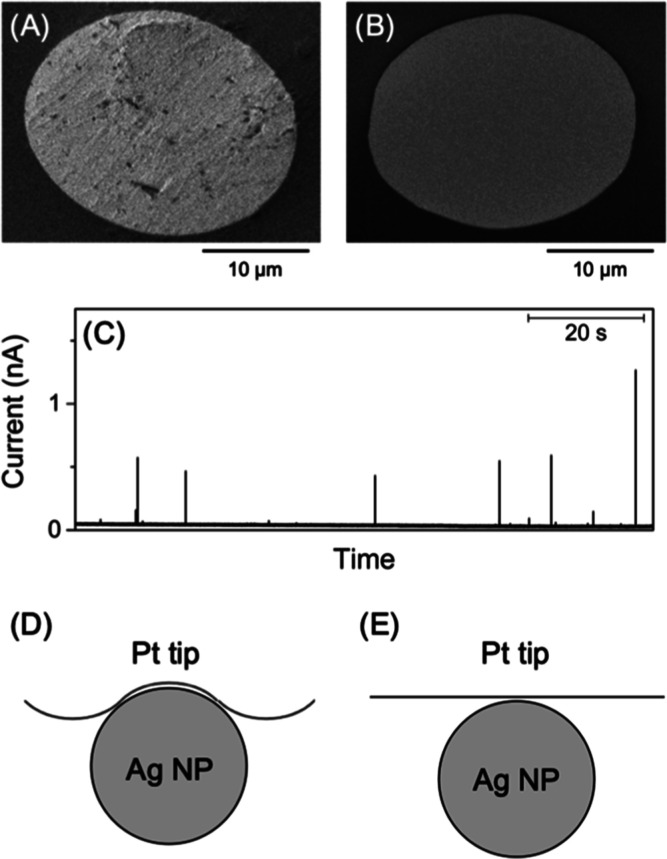
SEM images of (A) polished and (B) FIB-milled 25 μm-diameter Pt UMEs. Scale bars, 10 μm. (C) The current response of a FIB-milled 25 μm-diameter Pt UME to the oxidation of 40 nm-diameter citrate-capped Ag nanoparticles in the bulk solution. The solution of 47 pM nanoparticles also contained 20 mM KCl and 2 mM sodium citrate. (D) Scheme of fast and slow adsorption of Ag nanoparticles on the rough and flat surfaces of UME tips, respectively.

We propose that the rough surface of a mechanically polished tip serves as a template to efficiently adsorb Ag nanoparticles. The Pt tip was originally polished by using Al_2_O_3_ particles with diameters of ∼50 nm, which are comparable to those of Ag nanoparticles. Subsequently, Ag nanoparticles can be adsorbed through a large contact area with the rough Pt surface (Figure 6D). By contrast, the contact area is minimized when the tip surface is flattened by the FIB (Figure 6E). A similar template-based mechanism was demonstrated by electrochemically polymerizing 4-acetylbenzenediazonium on the template Ag nanoparticles adsorbed on the Au UME.^23^ A nanocavity was formed through the polymer layer by dissolving the nanoparticles to selectively adsorb nanoparticles of the same size as the templates. It should be noted that the template effect is minimal on the oxidation of the adsorbed nanoparticles. The average charges under spikes (0.22 ± 0.16 pC for 70 spikes) at the FIB-milled tip were similar to those observed with mechanically polished tips (0.23 ± 0.12 pC; see above). The charges correspond to 71% of the charges based on the complete oxidation of a 40 nm-diameter particle (0.31 pC).

### Slow Adsorption of PEG-Capped Ag Nanoparticles

We also investigated 40 nm-diameter PEG-capped Ag nanoparticles to find extremely slow adsorption on a 25 μm-diameter polished Pt tip. In this measurement, we employed a high concentration of 118 pM nanoparticles to observe a significant number of current spikes. Experimental collision frequencies of (1.6 ± 0.2) × 10^–1^ s^–1^ (*N* = 3) were obtained in the bulk solution (top panel of Figure 7). These frequencies were much lower than the diffusion-limited frequency of 47 s^–1^ in eq 1 with *D* = 1.2 × 10^–7^ cm^2^/s for 40 nm-diameter particles. The corresponding *f*_∞_/*f*_d_ value of 3.5 × 10^–3^ yields λ_ads_ = 3 × 10^–3^ (Figure 2), which is equivalent to *k*_ads_ = 3 × 10^–7^ cm/s. The λ_ads_ value is low enough to kinetically control the nanoparticle adsorption without any diffusional contribution (Figure 3). This prediction was confirmed by employing SECM. The UME tip was positioned at 3.2 μm from the glass substrate to observe the lack of a significant change in the collision frequency (bottom panel of Figure 7). Frequencies of (1.4 ± 0.1) × 10^–1^ s^–1^ (*N* = 3) at 3.2 μm from the substrate were not distinguishable from those in the bulk solution at the 95% confidence level. Average charges under spikes were also similarly low at 3.2 μm (0.05 ± 0.07 pC for 94 spikes) and in the bulk (0.07 ± 0.08 pC for 86 spikes). The respective averages correspond to 17 and 21% of the charges based on the complete oxidation of a 40 nm-diameter particle (0.31 pC). These average charges are significantly lower than those of citrate-capped nanoparticles (∼70% in Figure 4A). This result indicates that slow electron transfer through long PEG caps results in the oxidation of only ∼20% of Ag atoms before the Ag nanoparticles are desorbed from the Pt tip. The less complete oxidation of PEG-capped Ag nanoparticles is not due to faster nanoparticle desorption. The similar widths of current spikes (∼1 ms) between PEG- and citrate-capped Ag nanoparticles indicate similar residence times of the nanoparticles on the Pt tip.

**Figure 7:**
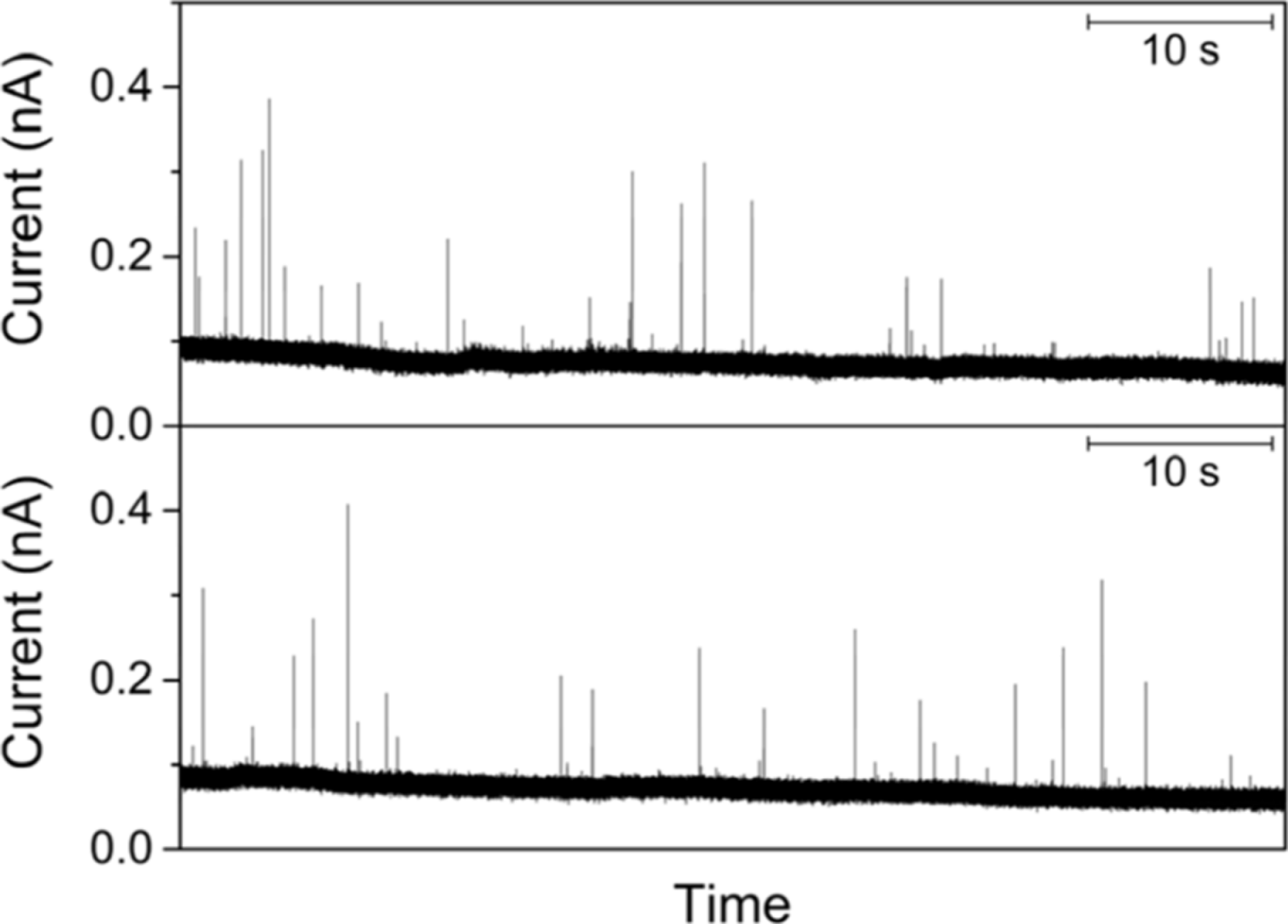
(A) The current response of a 25 μm-diameter Pt UME to the oxidation of 40 nm-diameter PEG-capped Ag nanoparticles in the bulk solution (top) and at *d* = 3.2 μm from the glass substrate (bottom). The solution of 118 pM nanoparticles also contained 20 mM KCl.

Intriguingly, *k*_ads_ varied by 4 orders of magnitude from the diffusion-limited adsorption of citrate-capped Ag nanoparticles to the purely kinetic adsorption of PEG-capped ones. The faster adsorption of citrate-capped Ag nanoparticles is attributed to the faster adsorption of citrate on the Pt tip. The fast and strong citrate adsorption on the Pt surface is driven by electron transfer^48,49^

8 $$Pt+{citrate}^{z-}⇄Pt-{citrate}_{ads}+ze^{-}$$

where *z* is the charge of citrate. The corresponding voltammetric response of oxidative citrate adsorption on the Pt(111) electrode has been observed at 0.3–0.5 V against RHE.^48,49^ These potentials are more negative than the tip potential applied for the oxidation of the Ag nanoparticles. Moreover, a tridentate citrate molecule may be adsorbed on both the Ag nanoparticle and the Pt surface for a faster UME–nanoparticle association. By contrast, PEG is oxidized but is not adsorbed on the Pt(111) surface biased at 0.3–0.5 V against RHE.^50^ The efficiency of oxidative citrate adsorption depends on the electrode material. Similar collision frequencies have been reported for citrate- and PEG-capped Ag nanoparticles at the carbon–fiber UME.^24^

## Conclusions

In this work, we applied stochastic SECM to kinetically investigate the adsorption-coupled oxidation of single Ag nanoparticles at the Pt UME tip. Theoretically, we modeled the steady-state diffusion, adsorption, and oxidation of Ag nanoparticles as an ensemble at the SECM tip near an insulating substrate to simulate the collision frequency of single nanoparticles. Experimentally, we demonstrated that SECM enables us to reliably and quantitatively resolve diffusion and adsorption steps from the fast oxidation of the adsorbed nanoparticles. Advantageously, SECM can be used to uniquely determine the adsorption rate constant and diffusion coefficient of a nanoparticle when the concentration of the nanoparticle is known. A reliable diffusion coefficient is needed to accurately determine the adsorption rate constant. Alternatively, the concentration of the nanoparticle can be determined by SECM when the diffusion coefficient of the nanoparticle is known. This advanced capability of SECM can be useful when standard methods of nanoparticle quantification are not applicable.^51^ SECM overcomes the current limitation of dominantly used amperometry, which requires both the diffusion coefficient and concentration of the nanoparticle to determine the adsorption rate constant.

We found that the adsorption rate constant can be nearly diffusion-controlled when a few crucial conditions are satisfied experimentally. Significantly, the collision frequency is directly related to the concentration of the nanoparticles and maximized under diffusion-controlled conditions to achieve the highest and most reproducible sensitivity for electroanalytical applications. Specifically, the nanoparticle solution must be clean to minimize the adventitious contamination of the electrode surface. Moreover, the surface of an UME tip must be roughened at the nanoscale to serve as a template for nanoparticle adsorption through a large contact area. Furthermore, capping reagents play a critical role in determining the kinetics of nanoparticle–electrode interactions. We propose the adsorption-coupled electron transfer^1–3^ of a capping reagent as a new covalent mechanism to accelerate nanoparticle adsorption for more sensitive and robust electrochemical detection.

## Supplementary Material



## Notes

SECM model, derivation of eq 6, range and distribution of nanoparticle diameters, SECM approach curve, cyclic voltammetry of polished and milled Pt tips, and COMSOL simulation report (PDF)
