# Supplementary material for: Adsorption-Coupled Oxidation of Single Ag Nanoparticles as Resolved by Stochastic Scanning Electrochemical Microscopy
Source: Anal Chem. 2026 Jan 2;98(1):611–8. doi: 10.1021/acs.analchem.5c05592 (PMC12809644; doi:10.1021/acs.analchem.5c05592)
Supplement: Supplementary file 1 [file ac5c05592_si_001.pdf]

Supporting Information

Adsorption-Coupled Oxidation of Single Ag Nanoparticles as Resolved by Stochastic Scanning Electrochemical Microscopy

Manu Jyothi Ravi, Donald C. Janda, Bagya Sivakumar, Aparajita Adak, and Shigeru Amemiya*

Department of Chemistry, University of Pittsburgh, 219 Parkman Avenue, Pittsburgh, Pennsylvania, 15260, United States

* To whom correspondence should be addressed. E-mail: amemiya@pitt.edu. Fax: 412-624-8611.

Table of Contents

SECM Model	S-2
Derivation of Eq 6	S-4
Range and Distribution of Nanoparticle Diameters	S-5
SECM Approach Curve	S-5
Cyclic Voltammetry of Polished and FIB-Milled Pt Tips	S-6
References	S-9
COMSOL Simulation Report	S-10

SECM Model. We quantitatively describe the SECM-based diffusion–reaction model based on the adsorption-coupled oxidation of Ag nanoparticles at the ultramicroelectrode (UME) tip. The diffusion problem is defined by setting up an SECM configuration in the cylindrical coordinates (Figure S-1). The problem is normalized and solved by employing COMSOL Multiphysics (version 6.2, COMSOL, Burlington, MA) as detailed below and summarized in the attached simulation report.

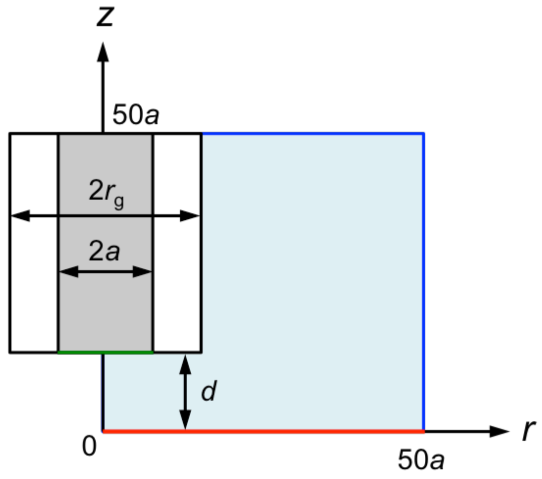

Figure S-1. Scheme of the SECM configuration with a glass-insulated Pt tip positioned over an insulating substrate. The red boundary represents the insulating substrate surface. The green boundary represents the tip surface. Black boundaries are insulating or a symmetry axis. Blue boundaries represent the bulk solution.

A steady-state diffusion equation for Ag nanoparticles is given in cylindrical coordinates by

$$\frac{\partial c_{\mathrm{NP}}}{\partial t} = D\left(\frac{\partial^2 c_{\mathrm{NP}}}{\partial r^2} + \frac{1}{r}\frac{\partial c_{\mathrm{NP}}}{\partial r} + \frac{\partial^2 c_{\mathrm{NP}}}{\partial z^2}\right) = 0 \qquad \text{(S-1)}$$

where c_{NP} and D are the concentration and diffusion coefficient of the nanoparticles, respectively. The tip reaction is controlled by the adsorption of Ag nanoparticles. The adsorbed nanoparticles are instantaneously oxidized at the tip, which is biased at a sufficiently positive potential. The corresponding boundary condition at the tip is given by

$$D\left(\frac{\partial c_{\mathrm{NP}}}{\partial z}\right)=-v_{\mathrm{tip}}=-k_{\mathrm{ads}}c_{\mathrm{NP}} \qquad \text{(S-2)}$$

where v_{tip} is the steady-state rate of nanoparticle adsorption on the tip and is independent of the tip potential, and k_{ads} is a rate constant for the adsorption of nanoparticles on the tip. Other boundary conditions are described in the caption of Figure S-1. Eq S-1 is solved to calculate the current response of the tip, i_{T}, as given by

$$i_{\mathrm{T}}=2\pi nF\int_0^a rv_{\mathrm{tip}}\mathrm{d}r \qquad \text{(S-3)}$$

where n is the number of electrons transferred from a nanoparticle to the tip. The tip current corresponds to the collision frequency of the nanoparticles as given by eq 4.

The diffusion equation for Ag nanoparticles (eq S-1) is defined and solved by using the following dimensionless parameters. Specifically, the diffusion equation in the dimensionless form is obtained from eq S-1 as

$$\frac{\partial^2 C_{\mathrm{NP}}}{\partial R^2}+\frac{1}{R}\frac{\partial C_{\mathrm{NP}}}{\partial R}+\frac{\partial^2 C_{\mathrm{NP}}}{\partial Z^2}=0 \qquad \text{(S-4)}$$

with

$$C_{\mathrm{NP}}=\frac{c_{\mathrm{NP}}}{c_0} \qquad \text{(S-5)}$$

$$R=\frac{r}{a} \qquad \text{(S-6)}$$

$$Z=\frac{z}{a} \qquad \text{(S-7)}$$

where c_0 is the bulk concentration of the nanoparticles and a is the tip radius. The boundary condition at the tip (eq S-2) is also normalized to yield

$$\left(\frac{\partial C_{\mathrm{NP}}}{\partial Z}\right) = -V_{\mathrm{tip}} = -\lambda_{\mathrm{ads}} C_{\mathrm{NP}} \qquad \text{(S-8)}$$

with

$$\lambda_{\mathrm{ads}} = \frac{k_{\mathrm{ads}} a}{D} \qquad \text{(S-9)}$$

Finally, a dimensionless tip current response, I_{T}, is given by normalizing eq S-3 to represent the dimensionless collision frequency, f/f_{∞}, as

$$I_{\mathrm{T}} = \frac{i_{\mathrm{T}}}{i_{\mathrm{T},\infty}} = \frac{f}{f_{\infty}} = 2\pi \int_0^1 R V_{\mathrm{tip}} \mathrm{d}R \qquad \text{(S-10)}$$

where $i_{\mathrm{T},\infty}$ is the tip current based on the diffusion-limited oxidation of Ag nanoparticles in the bulk solution and is given by eq 5, f and f_{∞} are collision frequencies of nanoparticles near the substrate and in the bulk solution, respectively, and x is a function of RG[S-1] (= r_{g}/a, where r_{g} is the outer radius of the UME tip).

Derivation of Eq 6. We generalized the original equation reported by Bard and co-workers[S-2] to derive eq 6 for an ultramicroelectrode with any RG in the bulk solution. Eq 6 describes the mixed kinetic and diffusion control of nanoparticle collision as represented by a steady-state concentration of the nanoparticle near the tip surface, c_{T}. Accordingly, a collision frequency, f_{∞}, is given by modifying eqs 1 and 2 as

$$f_{\infty} = 4xDa(c_0 - c_{\mathrm{T}}) \qquad \text{(S-11)}$$

$$f_{\infty} = \pi k_{\mathrm{ads}} a^2 c_{\mathrm{T}} \qquad \text{(S-12)}$$

A combination of eq S-11 with eq S-12 yields eq 6.

Range and Distribution of Nanoparticle Diameters. The diameters of Ag nanoparticles were determined by transmission electron microscopy as summarized in a histogram (Figure S-2). The average diameter of 40 ± 5 nm was determined for 100 nanoparticles.

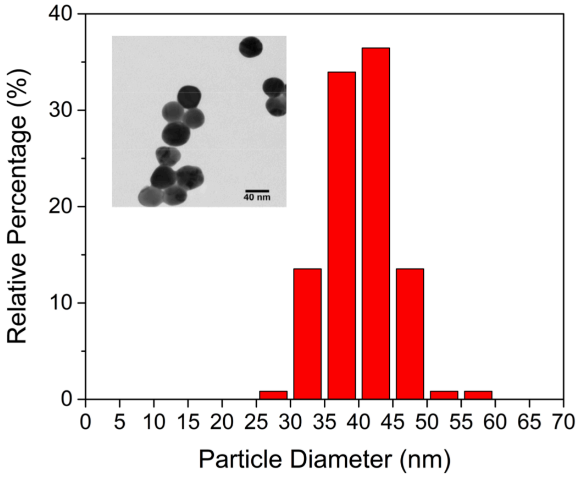

Figure S-2. Diameters of Ag nanoparticles determined by transmission electron microscopy.

SECM Approach Curve. The diffusion-limited oxygen reduction reaction (ORR) was driven at the tip with a Ag/AgCl reference/counter electrode to measure an approach curve for positioning the tip near the glass substrate. The cyclic voltammogram of ORR at the tip was measured to confirm a diffusion limit at < –0.4 V (Figure S-3A). The tip current based on the diffusion-limited ORR decreased as the tip approached a glass substrate (Figure S-3B), which hindered the diffusion of O_2 to the tip. The experimental approach curve fitted well with the theory of the negative feedback effect[S-1] to determine the tip–substrate distance.

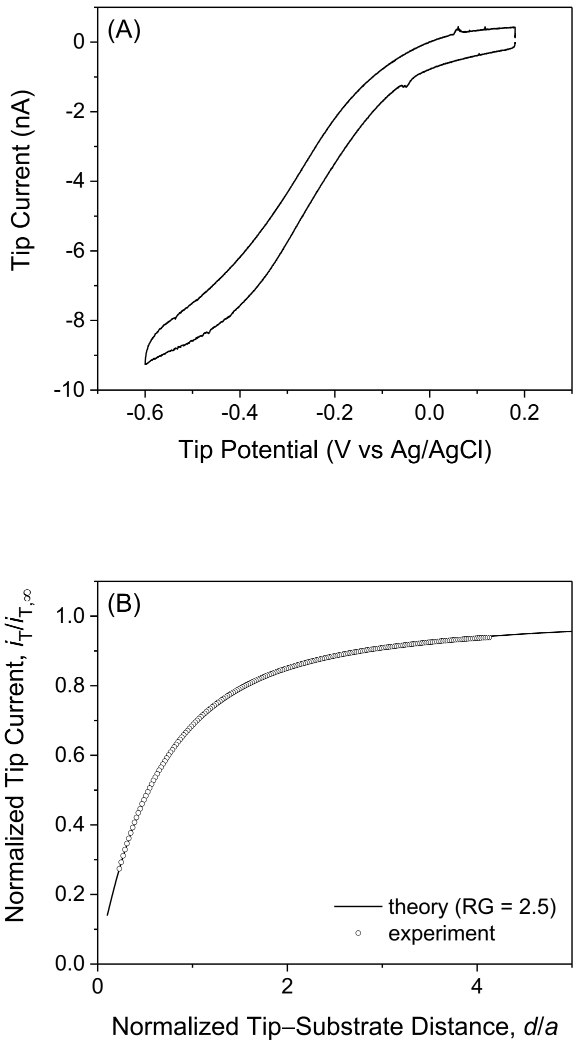

Figure S-3. (A) Cyclic voltammogram and (B) approach curve based on ORR at a 25µm-diameter polished Pt tip in the solution of 47 nM 40 nm-diameter citrate-capped Ag nanoparticles, 20 mM KCl, and 2 mM sodium citrate. Scan rate, 0.1 V/s in part (A).

Cyclic Voltammetry of Polished and FIB-Milled Pt Tips. We measured the voltammetric surface wave of underpotential hydrogen deposition at <0.35 V with polished and FIB-milled tips

(Figure S-4A and S-4B, respectively). The background current at >0.35 V was linearly extrapolated to estimate the charge under broad cathodic peaks as a measure of electrochemically active surface areas. The resultant charge density of 9.7×10^{-4} C/cm^2 at the polished tip was 4.6 times higher than that of 2.1×10^{-4} C/cm^2 at the FIB-milled tip. We also ensured that the surface of an FIB-milled Pt tip was active enough to efficiently mediate the hydrogen evolution reaction (Figure S-4C).

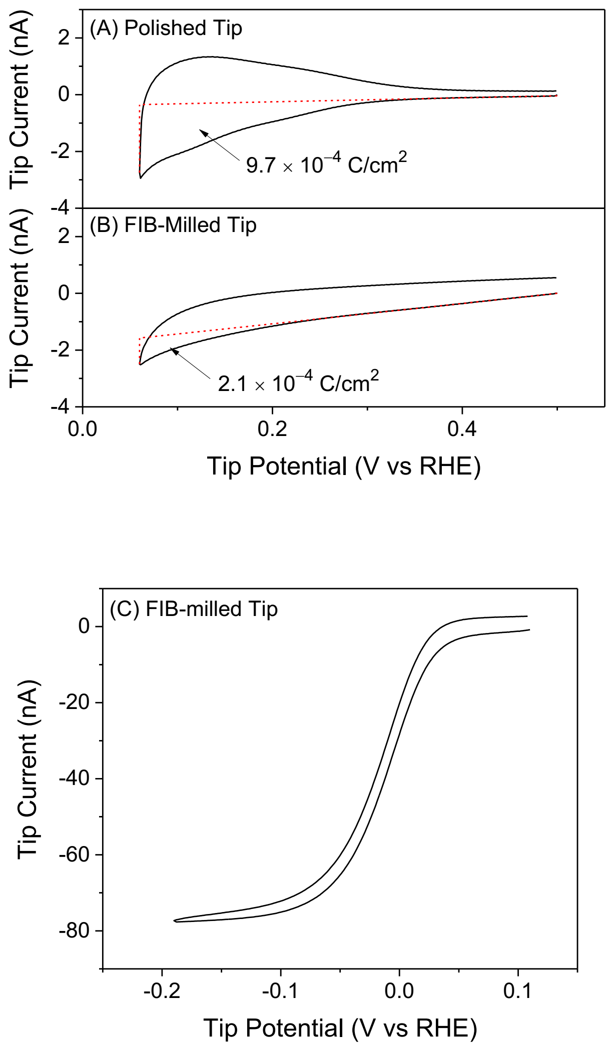

Figure S-4. Cyclic voltammograms of (A) polished and (B) FIB-milled 25μm-diameter Pt tips in 0.1 M $HClO_4$. Scan rate, 0.05 V/s. Charges surrounded by the forward wave and dotted red lines were integrated. (C) Cyclic voltammogram of a FIB-milled 25μm-diameter Pt tip in 1 mM $HClO_4$ and 0.1 M $KClO_4$. Scan rate, 0.05 V/s. Voltammograms were measured with a reversible hydrogen electrode (MiniHydroFlex, Biologic, Knoxville, TN).

REFERENCES

(S-1) Lefrou, C.; Cornut, R. Analytical expressions for quantitative scanning electrochemical microscopy (SECM). *ChemPhysChem* **2010**, *11*, 547–556.

(S-2) Kwon, S. J.; Zhou, H.; Fan, F.-R. F.; Vorobyev, V.; Zhang, B.; Bard, A. J. Stochastic electrochemistry with electrocatalytic nanoparticles at inert ultramicroelectrodes-theory and experiments. *Phys. Chem. Chem. Phys.* **2011**, *13* (12), 5394-5402.

ACET of Ag Nanoparticles

1 Global Definitions

Date	Aug 6, 2025, 5:47:56 PM

GLOBAL SETTINGS

Name	ACET of Ag Nanoparticles.mph
Path	E:\ACET of Ag Nanoparticles.mph
Version	COMSOL Multiphysics 6.2 (Build: 339)

USED PRODUCTS

COMSOL Multiphysics

COMPUTER INFORMATION

CPU	Intel64 Family 6 Model 158 Stepping 13, 8 cores, 15.79 GB RAM
Operating system	Windows 10

1.1 PARAMETERS

PARAMETERS 1

Name	Expression	Value	Description
lamdatip	1000 [m/s]	1000 m/s	
Nd	0.3 [m]	0.3 m	normalized tip-SiN distance
RG	2.5 [m]	2.5 m	normalized tip outer radius
c0	1 [mol/m^3]	1 mol/m^3	

2 Component 1

SETTINGS

Description	Value
Avoid inverted elements by curving interior domain elements	Off

2.1 DEFINITIONS

2.1.1 Probes

Boundary Probe 1

Probe type	Boundary probe

SELECTION

Geometric entity level	Boundary
Selection	Geometry geom1: Dimension 1: Boundary 3

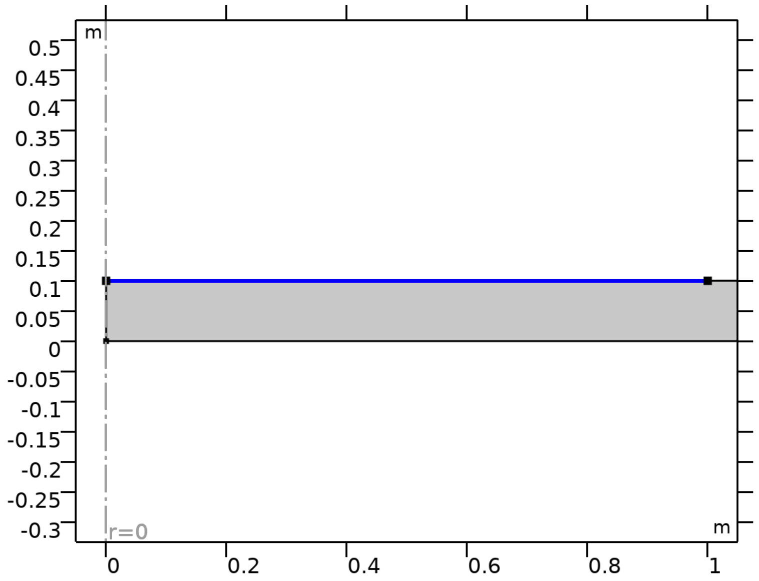

Selection

2.1.2 Nonlocal Couplings

Integration 1

Coupling type	Integration
Operator name	intop1

SELECTION

Geometric entity level	Boundary

Selection	Geometry geom1: Dimension 1: Boundary 3

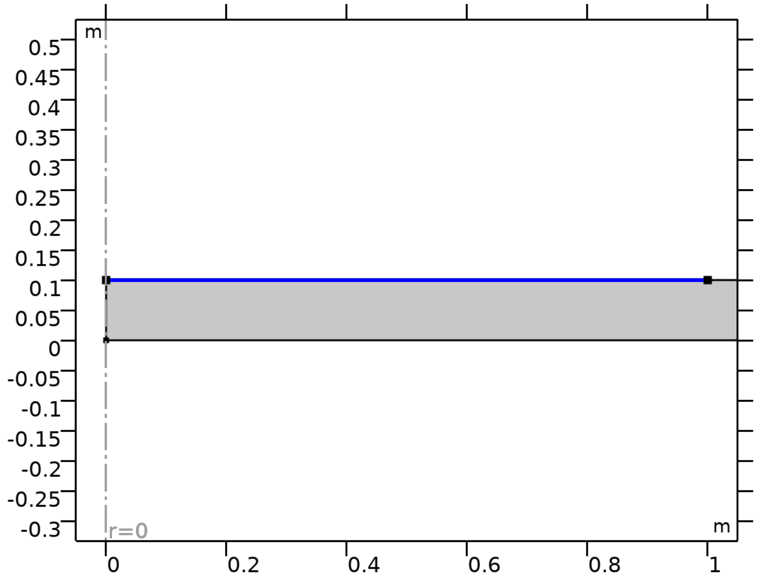

Selection

2.1.3 Coordinate Systems

Boundary System 1

Coordinate system type	Boundary system
Tag	sys1

COORDINATE NAMES

First	Second	Third
t1	to	n

2.2 GEOMETRY 1

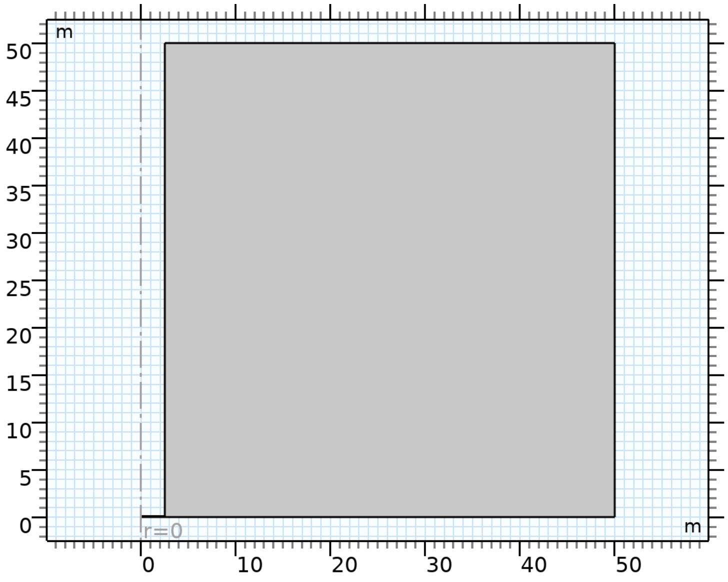

Geometry 1

UNITS

Length unit	m
Angular unit	deg

2.3 TRANSPORT OF DILUTED SPECIES

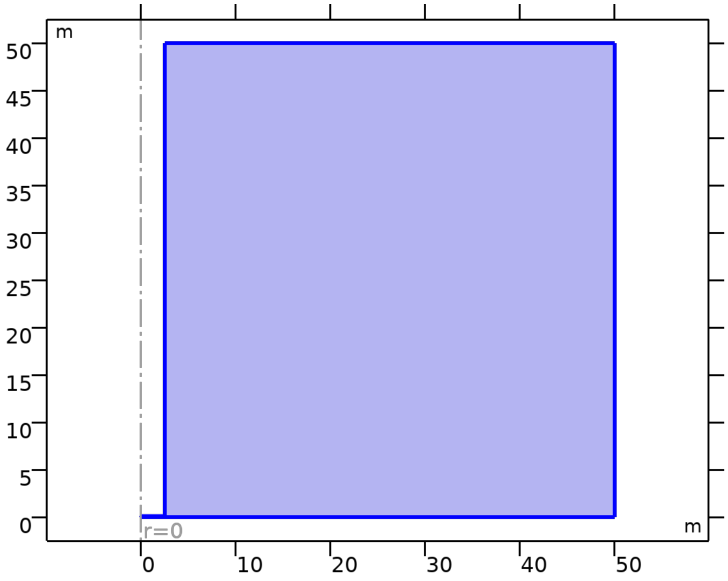

Transport of Diluted Species

EQUATIONS

$$\nabla \cdot \mathbf{J}_i + \mathbf{u} \cdot \nabla c_i = R_i$$

$$\mathbf{J}_i = -D_i \nabla c_i$$

FEATURES

Transport Properties 1	Domain
Axial Symmetry 1	Boundary
No Flux 1	Boundary
Initial Values 1	Domain
Concentration 1	Boundary
Flux 2	Boundary

2.4 MESH 1

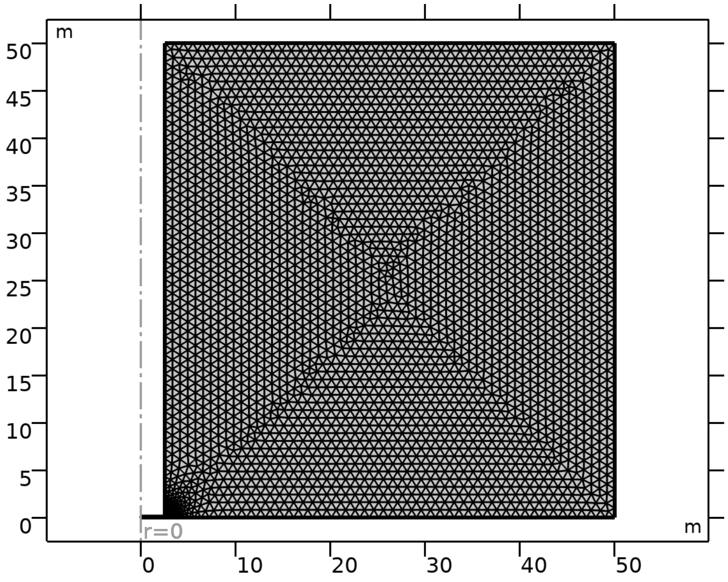

Mesh 1

3 Study 1

COMPUTATION INFORMATION

Computation time	1 min 14 s

3.1 PARAMETRIC SWEEP

Parameter name	Parameter value list	Parameter unit
Nd	range(10,-0.1,0.1)	m

STUDY SETTINGS

Description	Value
Sweep type	Specified combinations
Parameter name	Nd
Unit	m

PARAMETERS

Parameter name	Parameter value list	Parameter unit
Nd (normalized tip-SiN distance)	range(10,-0.1,0.1)	m

3.2 STATIONARY

STUDY SETTINGS

Description	Value
Include geometric nonlinearity	Off

PHYSICS AND VARIABLES SELECTION

Physics interface	Solve for	Equation form
Transport of Diluted Species (tds)	On	Automatic (Stationary)

STORE IN OUTPUT

Interface	Output	Selection
Transport of Diluted Species (tds)	Physics controlled	

MESH SELECTION

Component	Mesh
Component 1	Mesh 1

4 Results

4.1 DATA SETS

4.1.1 Study 1/Solution 1

SOLUTION

Description	Value
Solution	Solution 1 (sol1)
Component	Component 1 (comp1)

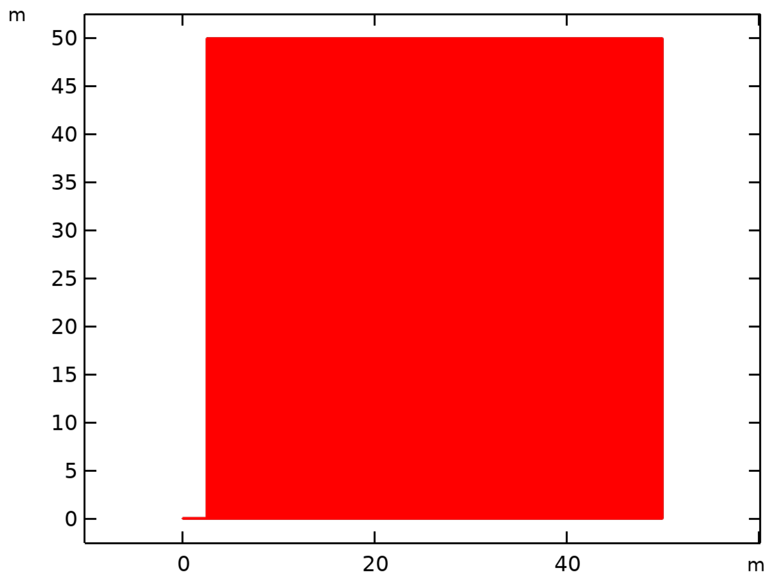

Dataset: Study 1/Solution 1

4.1.2 Revolution 2D

DATA

Description	Value
Dataset	Study 1/Solution 1 (sol1)

AXIS DATA

Description	Value
Axis entry method	Two points
Points	{{0, 0}, {0, 1}}

REVOLUTION LAYERS

Description	Value
Start angle	-90

Description	Value
Revolution angle	225

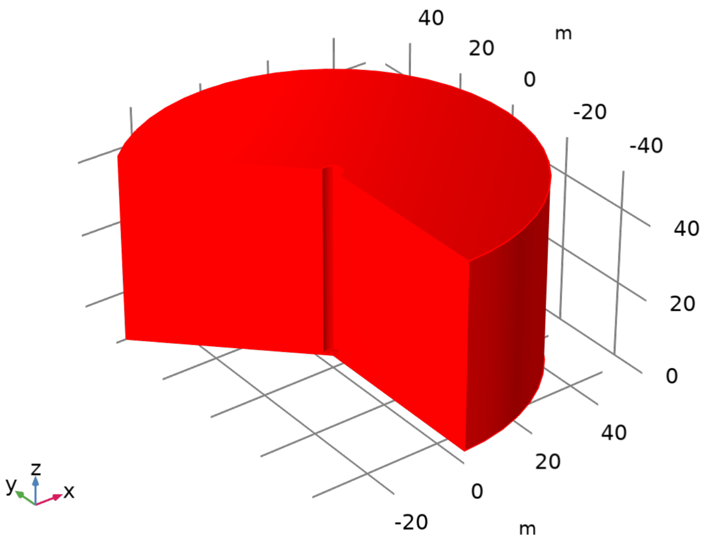

Dataset: Revolution 2D

4.1.3 Probe Solution 2

SOLUTION

Description	Value
Solution	Solution 1 (sol1)
Component	Component 1 (comp1)

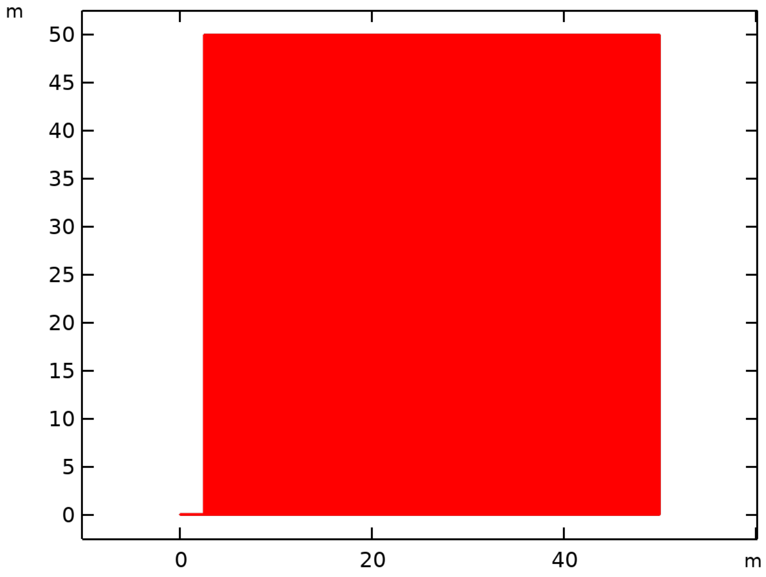

Dataset: Probe Solution 2

4.1.4 Boundary Probe 1

SELECTION

Geometric entity level	Boundary
Selection	Geometry geom1: Dimension 1: Boundary 3

DATA

Description	Value
Dataset	Probe Solution 2 (sol1)

SETTINGS

Description	Value
Method	Integration
Integration order	4
Integration order	On

4.1.5 Study 1/Parametric Solutions 1

SOLUTION

Description	Value
Solution	Parametric Solutions 1 (sol2)
Component	Component 1 (comp1)

4.1.6 Revolution 2D 2

DATA

Description	Value
Dataset	Study 1/Parametric Solutions 1 (sol2)

AXIS DATA

Description	Value
Axis entry method	Two points
Points	{{0, 0}, {0, 1}}

REVOLUTION LAYERS

Description	Value
Start angle	-90
Revolution angle	225

4.1.7 Study 1/Parametric Solutions 2

SOLUTION

Description	Value
Solution	Parametric Solutions 2 (sol53)
Component	Component 1 (comp1)

4.1.8 Revolution 2D 3

DATA

Description	Value
Dataset	Study 1/Parametric Solutions 2 (sol53)

AXIS DATA

Description	Value
Axis entry method	Two points
Points	{{0, 0}, {0, 1}}

REVOLUTION LAYERS

Description	Value
Start angle	-90
Revolution angle	225

4.1.9 Study 1/Parametric Solutions 3

SOLUTION

Description	Value
Solution	Parametric Solutions 3 (sol72)
Component	Component 1 (comp1)

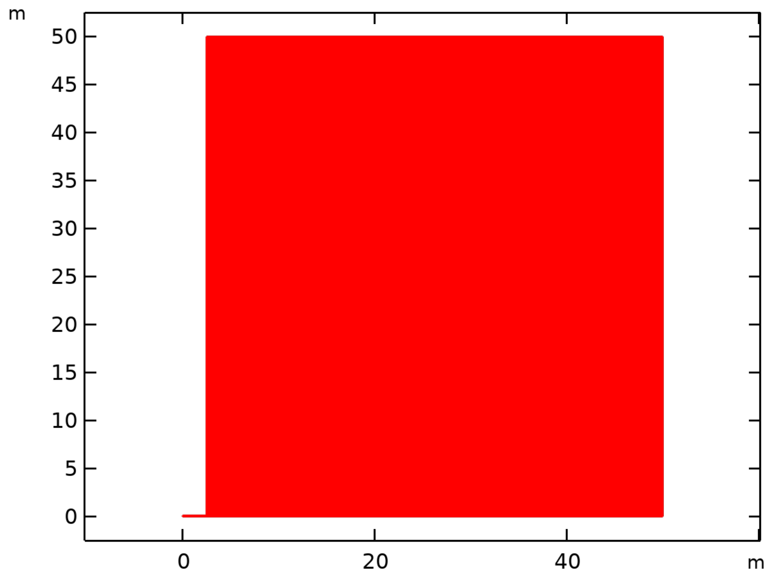

Dataset: Study 1/Parametric Solutions 3

4.1.10 Revolution 2D 4

DATA

Description	Value
Dataset	Study 1/Parametric Solutions 3 (sol72)

AXIS DATA

Description	Value
Axis entry method	Two points
Points	{{0, 0}, {0, 1}}

REVOLUTION LAYERS

Description	Value
Start angle	-90
Revolution angle	225

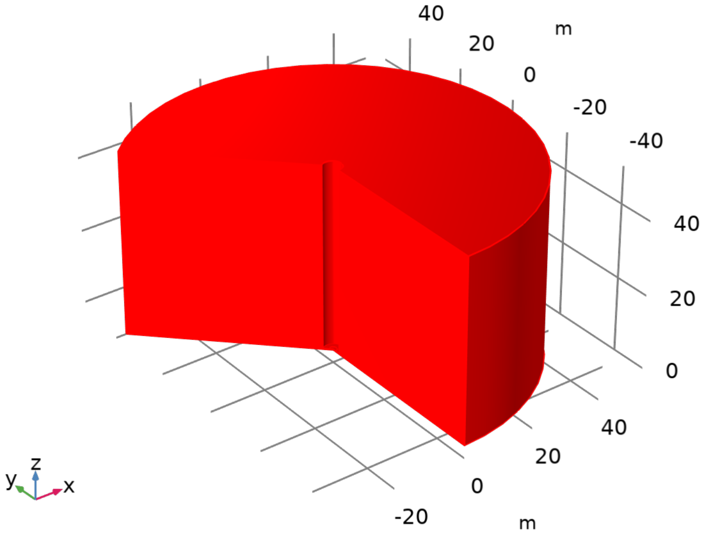

Dataset: Revolution 2D 4

4.2 DERIVED VALUES

4.2.1 Boundary Probe 1

OUTPUT

Evaluated in	Probe Table 1

DATA

Description	Value
Dataset	Boundary Probe 1

EXPRESSIONS

Expression	Unit	Description
intop1(tds.ndflux_c)	mol/s	

4.3 TABLES

4.3.1 Probe Table 1

Nd (m)	intop1(tds.ndflux_c) (mol/s), Boundary Probe 1
10	4.3071
9.9	4.3063
9.8	4.3051
9.7	4.3036
9.6	4.3028

Nd (m)	intop1(tds.ndflux_c) (mol/s), Boundary Probe 1
9.5	4.3018
9.4	4.3005
9.3	4.2988
9.2	4.298
9.1	4.2966
9	4.2953
8.9	4.2944
8.8	4.2928
8.7	4.2914
8.6	4.2902
8.5	4.2883
8.4	4.287
8.3	4.2854
8.2	4.2838
8.1	4.2826
8	4.281
7.9	4.279
7.8	4.2768
7.7	4.2754
7.6	4.2733
7.5	4.2719
7.4	4.2695
7.3	4.2675
7.2	4.2656
7.1	4.2634
7	4.2607
6.9	4.2586
6.8	4.2568
6.7	4.2535
6.6	4.2506
6.5	4.2485
6.4	4.246
6.3	4.2437
6.2	4.2397
6.1	4.2374

Nd (m)	intop1(tds.ndflux_c) (mol/s), Boundary Probe 1
6	4.2335
5.9	4.2304
5.8	4.2265
5.7	4.2227
5.6	4.2196
5.5	4.2154
5.4	4.211
5.3	4.2073
5.2	4.202
5.1	4.1974
5	4.1927
4.9	4.1876
4.8	4.182
4.7	4.1765
4.6	4.1707
4.5	4.1639
4.4	4.157
4.3	4.1508
4.2	4.143
4.1	4.1351
4	4.1272
3.9	4.1187
3.8	4.1097
3.7	4.0995
3.6	4.0891
3.5	4.0776
3.4	4.0662
3.3	4.0533
3.2	4.0397
3.1	4.0254
3	4.0091
2.9	3.9923
2.8	3.9736
2.7	3.9541
2.6	3.9322

Nd (m)	intop1(tds.ndflux_c) (mol/s), Boundary Probe 1
2.5	3.9088
2.4	3.883
2.3	3.8549
2.2	3.8237
2.1	3.7899
2	3.7524
1.9	3.7105
1.8	3.664
1.7	3.6121
1.6	3.5541
1.5	3.4888
1.4	3.415
1.3	3.3318
1.2	3.2364
1.1	3.1283
1	3.0049
0.9	2.8633
0.8	2.7007
0.7	2.5141
0.6	2.2991
0.5	2.0519
0.4	1.7664
0.3	1.4359
0.2	1.0491
0.1	0.58554

4.3.2 Evaluation 2D

Interactive 2D values

x	y	Value
2.8929	-0.21973	0.93422
0.33462	0.091346	-0.0086338
0.34174	0.012952	-0.086873
0.14762	0.68869	0.19022
0.73637	0.36524	0.92672
0.48924	0.66689	0.82101

x	y	Value
0.48924	0.67779	0.81734
0.54042	1.9754	0.024472
0.84368	0.1007	1.0062

4.4 PLOT GROUPS

4.4.1 Probe Plot Group 5

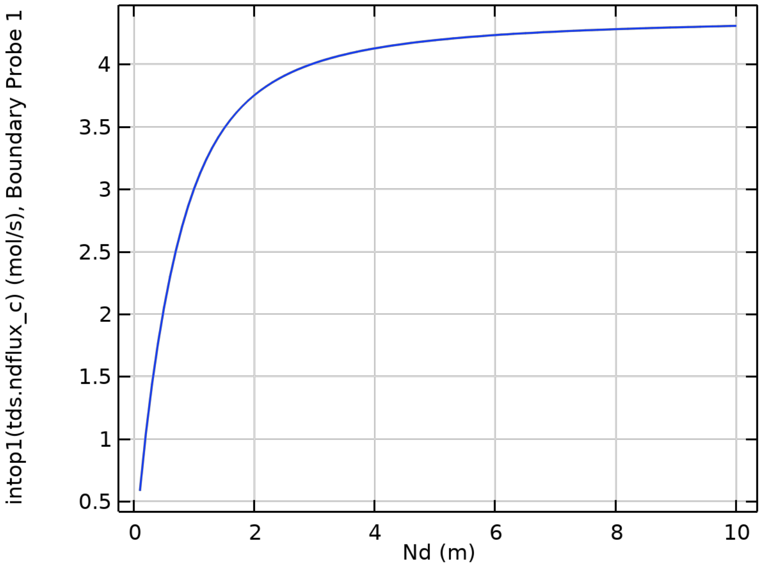

4.4.2 Concentration (tds)

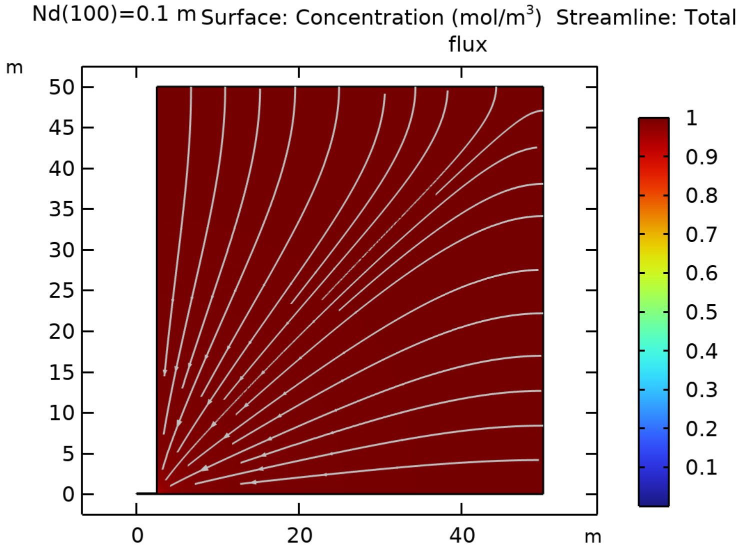

Surface: Concentration (mol/m^3) Streamline: Total flux

4.4.3 Concentration, 3D (tds)

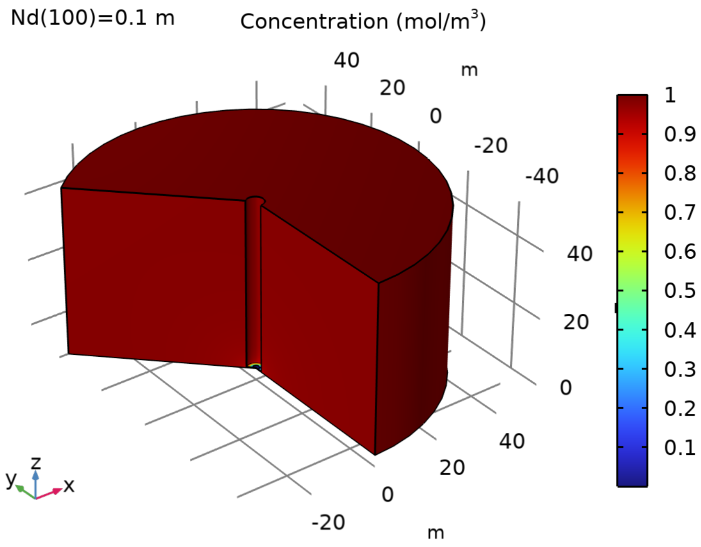

Concentration (mol/m³)
